# Projection-driven optimization framework for risk assessment of “dual carbon” audit in power grid enterprises with q-rung orthopair fuzzy MAGDM

**DOI:** 10.1016/j.heliyon.2024.e34020

**Published:** 2024-07-03

**Authors:** Shengnan Pan

**Affiliations:** Anhui Audit College, Hefei, 230601, China

**Keywords:** MAGDM, Q-ROFSs, Projection technique, Risk assessment of “dual carbon” audit, Power grid enterprises

## Abstract

Power grid enterprises are the backbone of promoting clean and low-carbon energy transformation, playing an important role in achieving carbon peak and carbon neutrality. It is very necessary to audit the implementation of the “dual carbon” work of power grid enterprises, in order to better implement the national “dual carbon” policy and serve the development of the national economy. The risk assessment of “dual carbon” audit in power grid enterprises is multiple-attribute group decision-making (MAGDM). In this study, in light with projection measure technique and bidirectional projection measure technique, four forms of projection measure technique with q-rung orthopair fuzzy sets (q-ROFSs) are conducted. Then, two weighed projection techniques are conducted to manage the MAGDM. Finally, a numerical example for risk assessment of “dual carbon” audit in power grid enterprises and comparative analysis is utilized to verify the developed techniques. The major contribution of this research is constructed: (1) entropy technique is implemented to determine the weight values in line with score number (SN) and accuracy number (AN); (2) two weighed projection techniques are implemented to put forward MAGDM with q-ROFSs; (3) the numerical example for risk assessment of “dual carbon” audit in power grid enterprises is implemented to show the two weighed projection techniques under q-ROFSs; and (4) comparative studies are constructed with existing techniques.

## 1 Introduction

In recent years, relevant departments have launched a series of innovative measures, including electricity marketization reform, carbon emission trading, and grid digitization, to establish a new type of power system and achieve the “dual carbon” goal [[1], [2], [3]]. In the process of promoting the market-oriented reform of electricity, the National Development and Reform Commission issued “Document No. 1439″ and “Document No. 809″ on October 11, 2021 and October 23, 2021, respectively, canceling the sales price of the industrial and commercial catalog, promoting all industrial and commercial users to enter the market, breaking the traditional situation of purchasing electricity solely through power grid enterprises, and stimulating the vitality of power supply services [[4], [5], [6]]. Regarding carbon emission trading, China officially launched the national carbon emission trading system in December 2017. Although the current focus is mainly on the power generation industry, State Grid is proactive and requires early carbon data analysis within enterprises [7]. At the same time, it actively provides carbon emission accounting and verification services, enters the secondary carbon emission market, develops carbon assets, and participates in carbon finance [8,9]. In addition, in promoting the digital transformation of the power grid, State Grid has also achieved significant results in the digitalization of power grid production, enterprise management, and customer service practices through the use of the “Cloud Big Things Mobile Intelligent Chain”. The Outline of the 14th Five Year Plan of the People's Republic of China lists “accelerating digital development and building a digital China” as a separate article, clearly stating that “digital transformation will drive the overall transformation of production methods, lifestyles, and governance methods.” On August 23, 2022, the State owned Assets Supervision and Administration Commission of the State Council issued the “Measures for Compliance Management of Central Enterprises,” which requires deepening the construction of state-owned enterprises under the rule of law, and promoting central enterprises to strengthen compliance management, ensure that the business management behavior and employee performance comply with legal provisions, industry standards, and relevant company bylaws, effectively preventing and controlling compliance risks [[10], [11], [12]]. In recent years, power grid enterprises have been committed to improving precise power services through digital technology, establishing a modern smart supply chain, achieving online processing of the entire business process, and meeting the diverse, personalized, and interactive needs of customers [13,14].

However, power grid enterprises also face certain legal risks in the process of promoting digital operations. For example, power data not only involves personal information such as user name, gender, home address, ID number and biometrics, but also may involve business secrets such as enterprise power data, business scale, customer information, etc [15]. If not properly managed, it is likely to cause large-scale infringement risk and even national network security systemic risk [[16], [17], [18], [19]]. Therefore, in order to prevent and resolve major business risks and promote the high-quality development of power grid enterprises, digital legal management must be placed in a more prominent position. In the process of building a new type of power system, power grid enterprises should prioritize planning, enhance their forward-looking awareness, systematically plan and formulate action plans for carbon peaking and carbon neutrality that are in line with the actual situation in the local area, providing scientific guidance and action guidelines for future local power grid construction [[20], [21], [22], [23]]. In the process of building a new type of power system, power grid enterprises should deepen the market-oriented reform of electricity, restore the commercialized nature of electricity, and promote the standardized and orderly entry of renewable energy into the market [24,25]. At the same time, it is necessary to prevent the risk of renewable energy consumption and conscientiously fulfill the obligation of fully guaranteeing the acquisition and consumption weight at the current stage [26,27]. Expanding green electricity trading, while ensuring energy security, planning and deploying entry into the carbon trading market, assisting the government in improving carbon trading policies and laws and regulations, actively developing renewable energy, and forming carbon asset projects [[28], [29], [30], [31]]. In the process of building a new type of power system, power grid enterprises should strengthen risk awareness, do a good job in digital compliance, strengthen power data management, and build a digital legal platform to prevent and resolve major business risks of the enterprise [[32], [33], [34]].

Multi-attribute decision-making (MADM), as important branch of modern decision science, is widely employed in management decision-making issues [[35], [36], [37], [38], [39], [40], [41]], such as investment project decision evaluation, supplier decision selection, and project site decision selection in real life. In practical life, MADM is mainly employed to divide into two main research parts [[42], [43], [44], [45]]: the first is to express expert MADM opinions and the second is full comparison and decision selection of various alternatives [46]. For first research part, during the research process of experts providing MADM opinions, the complexity external decision factors and human decision bounded rationality inevitably lead to fuzzy decision-making results. For this characteristic, more and more scholars generally employ fuzzy sets to obtain expert evaluation [47,48]. For the second part, scholars generally use the method of comparing and ranking schemes to select the optimal solution [49,50]. Motivated by the IFSs [51,52] and PFSs [53,54], Yager [55] conducted the q-ROFSs which is more suitable for managing the MAGDM. The different kinds of risk assessment of “dual carbon” audit in power grid enterprises are MAGDM. The q-ROFSs [55] are utilized as a technique for managing uncertain data during different kinds of risk assessment of “dual carbon” audit in power grid enterprises. The projection measure technique [56] and bidirectional projection measure technique [57] were utilized to handle the MAGDM. Until now, there is no efficient works conducted in line with projection techniques to cope with MAGDM with q-ROFSs. In this study, in light with projection measure technique and bidirectional projection measure technique, serval projection measure techniques with q-ROFS are conducted. Then, two weighed projection techniques are conducted to manage the MAGDM issue. Finally, a detailed example about risk assessment of “dual carbon” audit in power grid enterprises and comparative analysis is utilized to verify the developed techniques. The major research motivations of this research are managed: (1) entropy model is managed to determine the weight values in line with score number (SN) and accuracy number (AN); (2) two weighed projection techniques are implemented to cope with MAGDM under q-ROFSs; (3) the numerical example for risk assessment of “dual carbon” audit in power grid enterprises is implemented to show the two weighed projection techniques under q-ROFSs; and (4) comparative studies are managed with existing techniques.

To do so, the framework of this work is conducted. The q-ROFSs are conducted in Sect. 2. Sect. 3 conducts the projection technique and bidirectional projection technique under q-ROFSs. In Sect. 4, two projection measures techniques are conducted to cope with the MAGDM. In Sect. 5, numerical example for risk assessment of “dual carbon” audit in power grid enterprises and some comparative analysis is conducted. Sect. 6 conducted this research.

## 2 Preliminaries

The given q-ROFSs [55] is managed.

Definition 1 [55]. The q-ROFSs is built in Eq. (1):

(1) UU={⟨θ,(uα(θ),uβ(θ))⟩|θ∈Θ}

where the uα(θ),uβ(θ) is managed membership and non-membership, and in Eq. (2):

(2) $${\left(u\alpha \left(\theta \right)\right)}^{q}+{\left(u\beta \left(\theta \right)\right)}^{q}\leq 1,q\geq 1\text{.}$$

Definition 2 [55]**.** Let $uu_{1}=\left(u\alpha_{1},u\beta_{1}\right)$, $uu_{2}=\left(u\alpha_{2},u\beta_{2}\right)$, and uu=(uα,uβ) be q-ROFNs, then operational laws are built:

$$\begin{array}{c} \left(1\right)\;uu_{1}⊕uu_{2}=\left(\sqrt[{q}]{{\left(u\alpha_{1}\right)}^{q}+{\left(u\alpha_{2}\right)}^{q}-{\left(u\alpha_{1}\right)}^{q}\times {\left(u\alpha_{2}\right)}^{q}},u\beta_{1}\times u\beta_{2}\right)\text{;}\\ \left(2\right)\;uu_{1}⊗uu_{2}=\left(u\alpha_{1}\times u\alpha_{2},\sqrt[{q}]{{\left(u\beta_{1}\right)}^{q}+{\left(u\beta_{2}\right)}^{q}-{\left(u\beta_{1}\right)}^{q}{\left(u\beta_{2}\right)}^{q}}\right)\text{;}\\ \left(3\right)\;\lambda uu=\left(\sqrt[{q}]{1-{\left(1-u\alpha^{q}\right)}^{\lambda }},u\beta^{\lambda }\right),\lambda >0\text{;}\\ \left(4\right)\;{\left(uu\right)}^{\lambda }=\left(u\alpha^{\lambda },\sqrt[{q}]{1-{\left(1-u\beta^{q}\right)}^{\lambda }}\right),\lambda >0\text{;}\\ \left(5\right)\;uu^{c}=\left(u\beta ,u\alpha \right)\text{.} \end{array}$$

Definition 3 [55]**.** Let $uu_{1}=\left(u\alpha_{1},u\beta_{1}\right)$, $uu_{2}=\left(u\alpha_{2},u\beta_{2}\right)$, and uu=(uα,uβ), the operation laws are built.

$$\begin{array}{c} \left(1\right)\;uu_{1}⊕uu_{2}=uu_{2}⊕uu_{1},uu_{1}⊗uu_{2}=uu_{2}⊗uu_{1},{\left({\left(uu\right)}^{\lambda_{1}}\right)}^{\lambda_{2}}={\left(uu\right)}^{\lambda_{1}\lambda_{2}}\text{;}\\ \left(2\right)\;\lambda \left(uu_{1}⊕uu_{2}\right)=\lambda uu_{1}⊕\lambda \;uu_{2},{\left(uu_{1}⊗uu_{2}\right)}^{\lambda }={\left(uu_{1}\right)}^{\lambda }⊗{\left(uu_{2}\right)}^{\lambda }\text{;}\\ \left(3\right)\;\lambda_{1}uu⊕\lambda_{2}uu=\left(\lambda_{1}+\lambda_{2}\right)uu,{\left(uu\right)}^{\lambda_{1}}⊗{\left(uu\right)}^{\lambda_{2}}={\left(uu\right)}^{\left(\lambda_{1}+\lambda_{2}\right)}\text{.} \end{array}$$

Definition 4 [55,58]. Let uu=(uα,uβ) be q-ROFN, the score number (SN) and accuracy number (AN) are built in Eqs. (3) and (4):

(3) $$SN\left(dd\right)=\frac{1+{\left(u\alpha \right)}^{q}-{\left(u\beta \right)}^{q}}{2},SN\left(uu\right)\in \left[0,1\right]\text{.}$$

(4) $$AN\left(uu\right)=\frac{\left({\left(u\alpha \right)}^{q}+{\left(u\beta \right)}^{q}\right)}{2},AN\left(uu\right)\in \left[0,1\right]\text{.}$$

For two q-ROFNs $uu_{1}=\left(u\alpha_{1},u\beta_{1}\right)$ and $uu_{2}=\left(u\alpha_{2},u\beta_{2}\right)$, then (1) if $SN\left(uu_{1}\right)<SN\left(uu_{2}\right)\text{,}$ $uu_{1}<uu_{2}\text{;}$ (2) if $SN\left(uu_{1}\right)=SN\left(uu_{2}\right)\text{,}$ if $AN\left(uu_{1}\right)<AN\left(uu_{2}\right)\text{,}$ $uu_{1}<uu_{2}\text{;}$ if $AN\left(uu_{1}\right)=AN\left(uu_{2}\right)\text{,}$ $uu_{1}=uu_{2}$.

Definition 5 Let $uu_{1}=\left(u\alpha_{1},u\beta_{1}\right)$, $uu_{2}=\left(u\alpha_{2},u\beta_{2}\right)$, the inner product between $uu_{1}=\left(u\alpha_{1},u\beta_{1}\right)$ and $uu_{2}=\left(u\alpha_{2},u\beta_{2}\right)$ is managed in Eqs. (5), (6):

(5) $$uu_{1}\cdot uu_{2}=u\alpha_{1}^{q}\times u\alpha_{2}^{q}-u\alpha_{1}^{q}\times u\beta_{2}^{q}-u\alpha_{2}^{q}\times u\beta_{1}^{q}+3u\beta_{1}^{q}\times u\beta_{2}^{q}$$

(6) $$uu_{1}\cdot uu_{1}=u\alpha_{1}^{q}\times u\alpha_{1}^{q}-u\alpha_{1}^{q}\times u\beta_{1}^{q}-u\alpha_{1}^{q}\times u\beta_{1}^{q}+3u\beta_{1}^{q}\times u\beta_{1}^{q}={\left(u\alpha_{1}^{q}-u\beta_{1}^{q}\right)}^{2}+2{\left(d\beta_{1}^{q}\right)}^{2}$$

The useful q-ROFWA technique and q-ROFWG technique [58] is managed.

Definition 5 [58]**.** Let $uu_{j}=\left(u\alpha_{j},u\beta_{j}\right)$ with weight $uw={\left(uw_{1},uw_{2},\ldots ,uw_{n}\right)}^{T}$ which satisfies $uw_{j}>0$, $\sum_{j=1}^{n}uw_{j}=1$, then q-ROFWA model and q-ROFWG model are managed in Eqs. (7), (8):

(7) $$q-{\text{ROFWA}}_{uw}\left(uu_{1},uu_{2},\ldots ,uu_{n}\right)=\overset{n}{\underset{j=1}{⊕}}uw_{j}uu_{j}=\left(\sqrt[{q}]{1-\prod_{j=1}^{n}{\left(1-{\left(u\alpha_{j}\right)}^{q}\right)}^{uw_{j}}},\prod_{j=1}^{n}{\left(u\beta_{j}\right)}^{uw_{j}}\right)$$

and

(8) $$q-{\text{ROFWG}}_{uw}\left(uu_{1},uu_{2},\ldots ,uu_{n}\right)=\overset{n}{\underset{j=1}{⊗}}{\left(uu_{j}\right)}^{uw_{j}}=\left(\prod_{j=1}^{n}{\left(uu_{j}\right)}^{uw_{j}},\sqrt[{q}]{1-\prod_{j=1}^{n}{\left(1-{\left(u\beta_{j}\right)}^{q}\right)}^{uw_{j}}}\right)$$

## 3 Projection measures of q-ROFNs

Some projection measures are managed under q-ROFSs.

Definition 6 Let $uu_{1}=\left(u\alpha_{1},u\beta_{1}\right)$, $uu_{2}=\left(u\alpha_{2},u\beta_{2}\right)$ be q-ROFNs, the module of $uu_{1}=\left(u\alpha_{1},u\beta_{1}\right)$ and $uu_{2}=\left(u\alpha_{2},u\beta_{2}\right)$ could be managed in Eqs. (9), (10):

(9) $$\left\|uu_{1}\right\|=\sqrt{uu_{1}\cdot uu_{1}}=\sqrt{{\left(u\alpha_{1}^{q}-u\beta_{1}^{q}\right)}^{2}+2{\left(d\beta_{1}^{q}\right)}^{2}}$$

(10) $$\left\|uu_{2}\right\|=\sqrt{uu_{2}\cdot uu_{2}}=\sqrt{{\left(u\alpha_{2}^{q}-u\beta_{2}^{q}\right)}^{2}+2{\left(u\beta_{2}^{q}\right)}^{2}}$$

Definition 7 Let $uu_{1}=\left(u\alpha_{1},u\beta_{1}\right)$ and $uu_{2}=\left(u\alpha_{2},u\beta_{2}\right)$ be q-ROFNs, then the inner product $uu_{1}\cdot uu_{2}$ and cosine of included angle $\cos\left(uu_{1},uu_{2}\right)$ between $uu_{1}=\left(u\alpha_{1},u\beta_{1}\right)$ and $uu_{2}=\left(u\alpha_{2},u\beta_{2}\right)$ are denoted in Eq. (11), (12):

(11) $$uu_{1}\cdot uu_{2}=u\alpha_{1}^{q}\times u\alpha_{2}^{q}-u\alpha_{1}^{q}\times u\beta_{2}^{q}-u\alpha_{2}^{q}\times u\beta_{1}^{q}+3u\beta_{1}^{q}\times u\beta_{2}^{q}$$

(12) $$\cos\left(uu_{1},uu_{2}\right)=\frac{uu_{1}.uu_{2}}{\left\|uu_{1}\right\|\left\|uu_{2}\right\|}=\frac{u\alpha_{1}^{q}\times u\alpha_{2}^{q}-u\alpha_{1}^{q}\times u\beta_{2}^{q}-u\alpha_{2}^{q}\times u\beta_{1}^{q}+3u\beta_{1}^{q}\times u\beta_{2}^{q}}{\sqrt{{\left(u\alpha_{1}^{q}-u\beta_{1}^{q}\right)}^{2}+2{\left(u\beta_{1}^{q}\right)}^{2}}\cdot \sqrt{{\left(u\alpha_{2}^{q}-u\beta_{2}^{q}\right)}^{2}+2{\left(u\beta_{2}^{q}\right)}^{2}}}$$

Definition 8 Assume that $UU_{i}=\left(uu_{i1},uu_{i2},\ldots ,uu_{in}\right)\left(i=1,2,\ldots ,m\right)$ and $UU=\left(uu_{1},uu_{2},\ldots ,uu_{n}\right)$ be ideal alternative with q-ROFNs, where $uu_{ij}=\left(u\alpha_{ij},u\beta_{ij}\right),i=1,2,\ldots ,m,j=1,2,\ldots ,n$ and $uu_{j}=\left(u\alpha_{j},u\beta_{j}\right),j=1,2,\ldots ,n\text{.}$ Then the projection of vector $UU_{i}$ on UU could be managed in Eq. (13), (14):

(13) $$\mathrm{PR}\;J_{UU}\left(UU_{i}\right)=\left\|UU_{i}\right\|\sum_{j=1}^{n}\cos\left(UU_{i},UU\right)=\left\|UU_{i}\right\|\cdot \frac{\sum_{j=1}^{n}\left(uu_{ij}^{q}uu_{j}^{q}+uu_{ij}^{q}uu_{j}^{q}\right)}{\left\|UU_{i}\right\|\left\|UU\right\|}=\frac{1}{\left\|UU\right\|}\sum_{j=1}^{n}\left(u\alpha_{ij}^{q}\times u\alpha_{j}^{q}-u\alpha_{ij}^{q}\times u\beta_{j}^{q}-u\alpha_{j}^{q}\times u\beta_{j}^{q}+3u\beta_{ij}^{q}\times u\beta_{j}^{q}\right)$$

where

(14) $$\left\|UU\right\|=\sqrt{\sum_{j=1}^{n}\left({\left(u\alpha_{j}^{q}-u\beta_{j}^{q}\right)}^{2}+2{\left(u\beta_{j}^{q}\right)}^{2}\right)}$$

Obviously, the greater $\mathrm{PR}\;J_{UU}\left(UU_{i}\right)$, the closer $DD_{i}$ to DD, the better $DD_{i}$ is.

In practical MADM, the weight values are important factors should be considered, thus, let $dw=\left(dw_{1},dw_{2},\ldots ,dw_{n}\right)$ be the weights, the given weighted projection of $DD_{i}$ on DD could be managed.

Definition 9 Let $UU_{i}=\left(uu_{i1},uu_{i2},\ldots ,uu_{in}\right)\left(i=1,2,\ldots ,m\right)$ and $UU=\left(uu_{1},uu_{2},\ldots ,uu_{n}\right)$ be ideal alternative with weight $uw=\left(uw_{1},uw_{2},\ldots ,uw_{n}\right)$, where $uu_{ij}=\left(u\alpha_{ij},u\beta_{ij}\right),i=1,2,\ldots ,m,j=1,2,\ldots ,n$ and $uu_{j}=\left(u\alpha_{j},u\beta_{j}\right),j=1,2,\ldots ,n\text{.}$ Then the weighted projection of $UU_{i}$ on UU could be managed in Eq. (15), (16):

(15) $$WPRJ_{UU}\left(UU_{i}\right)={\left\|UU_{i}\right\|}_{uw}\sum_{j=1}^{n}\cos\;{\left(UU_{i},UU\right)}_{uw}={\left\|UU_{i}\right\|}_{dw}\cdot \frac{\sum_{j=1}^{n}uw_{j}^{2q}\left(u\alpha_{ij}^{q}\times u\alpha_{j}^{q}-u\alpha_{ij}^{q}\times u\beta_{j}^{q}-u\alpha_{j}^{q}\times u\beta_{j}^{q}+3u\beta_{ij}^{q}\times u\beta_{j}^{q}\right)}{{\left\|UU_{i}\right\|}_{dw}{\left\|UU\right\|}_{dw}}=\frac{1}{{\left\|UU\right\|}_{dw}}\sum_{j=1}^{n}dw_{j}^{2q}\left(u\alpha_{ij}^{q}\times u\alpha_{j}^{q}-u\alpha_{ij}^{q}\times u\beta_{j}^{q}-u\alpha_{j}^{q}\times u\beta_{j}^{q}+3u\beta_{ij}^{q}\times u\beta_{j}^{q}\right)$$

where

(16) $${\left\|UU\right\|}_{uw}=\sqrt{\sum_{j=1}^{n}\left({\left(uw_{j}\left(u\alpha_{j}^{q}-u\beta_{j}^{q}\right)\right)}^{2}+3{\left(uw_{j}u\beta_{j}^{q}\right)}^{2}\right)}$$

where $uw_{j}\left(j=1,2,\ldots ,n\right)$ meets $0\leq uw_{j}\leq 1,\sum_{j=1}^{n}uw_{j}=1\text{.}$ Obviously, the greater value $WPRJ_{UU}\left(UU_{i}\right)$, the closer $UU_{i}$ to UU, which is the better alternatives $UU_{i}$.

The bidirectional projection measure technique with q-ROFNs are conducted.

Definition 10 Let $UU_{i}=\left(uu_{i1},uu_{i2},\ldots ,uu_{in}\right)\left(i=1,2,\ldots ,m\right)$ and $UU=\left(uu_{1},uu_{2},\ldots ,uu_{n}\right)$ be ideal alternative with weight $uw=\left(uw_{1},uw_{2},\ldots ,uw_{n}\right)$, where $uu_{ij}=\left(u\alpha_{ij},u\beta_{ij}\right),i=1,2,\ldots ,m,j=1,2,\ldots ,n$ and $uu_{j}=\left(u\alpha_{j},u\beta_{j}\right),j=1,2,\ldots ,n\text{.}$ The bidirectional projection of $UU_{i}$ on UU could be managed in Eq. (17), (18), (20):

(17) $$BPROJ\left(UU_{i},UU\right)=\frac{1}{1+\left|\frac{UU_{i}\cdot UU}{\left\|UU_{i}\right\|}-\frac{UU_{i}\cdot UU}{\left\|UU\right\|}\right|}=\frac{\left\|UU_{i}\right\|\left\|UU\right\|}{\left\|UU_{i}\right\|\left\|UU\right\|+\left|\left\|UU_{i}\right\|-\left\|UU\right\|\right|\left(UU_{i}\cdot UU\right)}$$

where

(18) $$\left\|UU\right\|=\sqrt{\sum_{j=1}^{n}\left({\left(u\alpha_{j}^{q}-u\beta_{j}^{q}\right)}^{2}+2{\left(u\beta_{j}^{q}\right)}^{2}\right)}$$

(19) $$\left\|UU_{i}\right\|=\sqrt{\sum_{j=1}^{n}\left({\left(u\alpha_{ij}^{q}-u\beta_{ij}^{q}\right)}^{2}+2{\left(u\beta_{ij}^{q}\right)}^{2}\right)}$$

(20) $$\left(UU_{i}\cdot UU\right)=\sum_{j=1}^{n}\left(u\alpha_{ij}^{q}\times u\alpha_{j}^{q}-u\alpha_{ij}^{q}\times u\beta_{j}^{q}-u\alpha_{j}^{q}\times u\beta_{j}^{q}+3u\beta_{ij}^{q}\times u\beta_{j}^{q}\right)$$

Obviously, the greater $BPROJ\left(UU_{i},UU\right)$, the closer vector $UU_{i}$ to UU, which indicates the better the $UU_{i}$.

Consider the decision weight values of q-ROFNs, weighted bidirectional projection technique of vector $UU_{i}$ on UU could be managed.

Definition 11 Let $UU_{i}=\left(uu_{i1},uu_{i2},\ldots ,uu_{in}\right)\left(i=1,2,\ldots ,m\right)$ and $UU=\left(uu_{1},uu_{2},\ldots ,uu_{n}\right)$ be the ideal alternative, where $uu_{ij}=\left(u\alpha_{ij},u\beta_{ij}\right),i=1,2,\ldots ,m,j=1,2,\ldots ,n$ and $uu_{j}=\left(u\alpha_{j},u\beta_{j}\right),j=1,2,\ldots ,n\text{.}$ The weighted bidirectional projection of $UU_{i}$ on UU could be built in Eq. (21), (22), (23), (24):

(21) $$WBPROJ{\left(UU_{i},UU\right)}_{uw}=\frac{1}{1+\left|\frac{{\left(UU_{i}\cdot UU\right)}_{uw}}{{\left\|UU_{i}\right\|}_{uw}}-\frac{{\left(UU_{i}\cdot UU\right)}_{uw}}{{\left\|UU\right\|}_{uw}}\right|}=\frac{{\left\|UU_{i}\right\|}_{uw}{\left\|UU\right\|}_{uw}}{{\left\|UU_{i}\right\|}_{uw}{\left\|UU\right\|}_{uw}+\left|{\left\|UU_{i}\right\|}_{uw}-{\left\|UU\right\|}_{uw}\right|{\left(UU_{i}\cdot UU\right)}_{uw}}$$

where

(22) $${\left\|UU\right\|}_{uw}=\sqrt{\sum_{j=1}^{n}\left({\left(uw_{j}\left(u\alpha_{j}^{q}-u\beta_{j}^{q}\right)\right)}^{2}+2{\left(uw_{j}u\beta_{j}^{q}\right)}^{2}\right)}$$

(23) $${\left\|UU_{i}\right\|}_{dw}=\sqrt{\sum_{j=1}^{n}\left({\left(uw_{j}\left(u\alpha_{ij}^{q}-u_{ij}^{q}\right)\right)}^{2}+2{\left(uw_{j}u\beta_{ij}^{q}\right)}^{2}\right)}$$

(24) $${\left(UU_{i}\cdot UU\right)}_{dw}=\sum_{j=1}^{n}uw_{j}^{2q}\left(u\alpha_{ij}^{q}\times u\alpha_{j}^{q}-u\alpha_{ij}^{q}\times u\beta_{j}^{q}-u\alpha_{j}^{q}\times u\beta_{j}^{q}+3u\beta_{ij}^{q}\times u\beta_{j}^{q}\right)$$

where $uw_{j}\left(j=1,2,\ldots ,n\right)$ satisfies $0\leq uw_{j}\leq 1,\sum_{j=1}^{n}uw_{j}=1\text{.}$ Obviously, the greater value $WBPRJ_{UU}\left(UU_{i}\right)$, the closer $UU_{i}$ to UU, which is better alternative $UU_{i}$.

## 4 Projection technique with q-ROFNs

Suppose that m alternatives $UA=\left\{UA_{1},UA_{2},\ldots ,UA_{m}\right\}$, n attributes $UG=\left\{UG_{1},UG_{2},\ldots ,UG_{n}\right\}$ with weight $uw=\left\{uw_{1},uw_{2},\ldots ,uw_{n}\right\}$ and λ experts $UD=\left\{UD_{1},UD_{2},\ldots ,UD_{t}\right\}$ with weight $u\omega =\left\{u\omega_{1},u\omega_{2},\ldots u\omega_{\lambda }\right\}$ which satisfy $0\leq uw_{j}\leq 0,0\leq u\omega_{k}\leq 0$ and $\sum_{j=1}^{n}uw_{j}=1,\sum_{k=1}^{\lambda }u\omega_{k}=1$. The assessed matrix are managed through q-ROFNs $UR^{\left(k\right)}={\left[UR_{ij}^{\left(k\right)}\right]}_{m\times n}=\left(u\alpha_{ij}^{\left(k\right)},u\beta_{ij}^{\left(k\right)}\right)u\alpha_{ij}^{\left(k\right)}\in \left[0,1\right]$ is membership of $UA_{i}$ meets $UG_{j}$ utilized by DM, $u\beta_{ij}^{\left(k\right)}\in \left[0,1\right]$ is non-membership of $UA_{i}$ don't meet $UG_{j}$ managed, then the steps of projection technique under q-ROFNs is conducted.

Step 1 Conduct the matrix $UR^{\left(k\right)}={\left[UR_{ij}^{\left(k\right)}\right]}_{m\times n}=\left(u\alpha_{ij}^{\left(k\right)},u\beta_{ij}^{\left(k\right)}\right)$ with q-ROFNs.

Step 2 In line with q-ROFWA technique, the $UR_{ij}^{\left(k\right)}$ is utilized to obtain $UR_{ij}$ and $UR_{i}={\left(UR_{ij}\right)}_{m\times n}={\left(u\alpha_{ij},u\beta_{ij}\right)}_{m\times n}$;

Step 3 Conduct the attribute weight through entropy.

Entropy [59] is conventional technique to obtain weight values. The normalized decision matrix $NUM_{ij}$ is managed in Eq. (25):

(25) $$NUM_{ij}=\frac{1}{2}\left(\frac{1+SN\left(u\alpha_{ij},u\beta_{ij}\right)}{\sum_{i=1}^{m}\left(1+\left(SN\left(u\alpha_{ij},u\beta_{ij}\right)\right)\right)}+\frac{1+AN\left(u\alpha_{ij},u\beta_{ij}\right)}{\sum_{i=1}^{m}\left(1+\left(AN\left(u\alpha_{ij},u\beta_{ij}\right)\right)\right)}\right)\text{,}$$

The q-ROFS Shannon entropy $q-ROFSSE=\left(q-ROFSSE_{1},q-ROFSSE_{2},\cdots ,q-ROFSSE_{n}\right)$ is managed in Eq. (26):

(26) $$q-ROFSSE_{j}=-\frac{1}{\ln\;m}\sum_{i=1}^{m}NUM_{ij}\;\ln\;NUM_{ij}$$

and $NUM_{ij}\;\ln\;NUM_{ij}=0$ if $NUM_{ij}=0$.

Then, the weight value $uw=\left(uw_{1},uw_{2},\cdots ,uw_{n}\right)$ is managed in Eq. (27):

(27) $$uw_{j}=\frac{1-q-ROFSSE_{j}}{\sum_{j=1}^{n}\left(1-q-ROFSSE_{j}\right)},j=1,2,\cdots ,n\text{.}$$

Step 4 Conduct the ideal information alternative $UR^{+}=\left(UR_{j}^{+}\right)\left(j=1,2,\cdots ,n\right)$ in Eq. (28).

(28) $$SN\left(UR_{j}^{+}\right)=SN\left(u\alpha_{j}^{+},u\beta_{j}^{+}\right)=\max_{i}SN\left(UR_{ij}\right)=\max_{i}SN\left(u\alpha_{ij},u\beta_{ij}\right)$$

Step 5 Conduct the $WPRJ\left(UR_{i},UR^{+}\right)$ and $WBPRJ\left(UR_{i},UR^{+}\right)$ between $UR_{i}$ and $UR^{+}$ according to equations (15), (21) to sort the alternatives.

Step 6 In line with $WPRJ\left(UR_{i},UR^{+}\right)$ and $WBPRJ\left(UR_{i},UR^{+}\right)$, the greater measures is better alternative $UA_{i}$.

## 5 Numerical example and comparative analysis

### 5.1 Numerical example

With the increasing severity of global climate change, countries have put forward carbon neutrality goals, requiring the energy industry to reduce carbon emissions and achieve sustainable development. In this context, power grid enterprises, as a core component of energy distribution and transmission, bear important responsibilities. How to achieve transformation and improve energy efficiency under the pressure of “dual carbon” has become an urgent problem for power grid enterprises. The importance of carbon neutrality goals for power grid enterprises cannot be underestimated. Global climate change has become an urgent challenge facing the world today, requiring active action to reduce carbon emissions and curb global temperature rise. In this context, countries have proposed carbon neutrality goals with the aim of achieving net zero carbon emissions. For power grid enterprises, this means reducing the carbon emissions they generate during energy distribution and transmission. Power grid enterprises are an important component of the energy supply chain, and their activities have a direct impact on carbon emissions. Traditional power systems may rely on fossil fuels, which can lead to significant carbon dioxide emissions. In order to achieve carbon neutrality goals, power grid enterprises need to take a series of measures, including gradually phasing out high carbon energy, improving energy efficiency, and adopting renewable energy. In addition, power grid enterprises also bear important social responsibilities. They not only need to meet energy needs, but also need to ensure the reliability and stability of electricity supply to maintain the economic operation of the country and region. Through the transformation of the digital economy, power grid enterprises can better manage energy distribution, achieve intelligent operation, improve power supply quality, and reduce carbon emissions. The goal of carbon neutrality is not only a moral responsibility for power grid enterprises to address climate change, but also a business opportunity. Through the transformation of the digital economy, power grid enterprises can improve their competitiveness and sustainability while achieving carbon neutrality goals, making positive contributions to a clean and sustainable future energy system. The risk assessment of “dual carbon” audit in power grid enterprises is MADM. In this work, a numerical example is provided for risk assessment of “dual carbon” audit in power grid enterprises by employing developed projection techniques under q-ROFNs. The five power grid enterprises $UA_{i}\left(i=1,2,3,4,5\right)$ to be selected in line with six attributes: ① $UG_{1}$ is carbon asset management capability; ② $UG_{2}$ is carbon market construction and operation; ③ $UG_{3}$ is electricity revenue; ④ $UG_{4}$ is annual carbon reduction achieved by the power grid; ⑤ $UG_{5}$ is renewable energy consumption capacity; ⑥ $UG_{6}$ is power transmission capacity of cross provincial and cross regional transmission channels. The five power grid enterprises $UA_{i}\left(i=1,2,3,4,5\right)$ are assessed through employing linguistic scale (See Table 1 [60]) in line with six decision attributes through inviting three experts $UR^{\left(k\right)}$ with equal weight information uω=(1/3,1/3,1/3).

**Table 1:** Linguistic scales and q-ROFNs.

Linguistic scales	q-ROFNs
Exceedingly Terrible-UET	(0.10,0.80)
Very Terrible-UVT	(0.20,0.70)
Terrible-UT	(0.30,0.60)
Medium-UM	(0.50,0.50)
Well-UW	(0.65,0.30)
Very Well-UVW	(0.75,0.20)
Exceedingly Well-UEW	(1.0,0.0)

Projection measure techniques with q-ROFNs is utilized to manage the risk assessment of “dual carbon” audit in power grid enterprises.

Step 1 Conduct the matrix $UR^{\left(t\right)}={\left[UR_{ij}^{\left(t\right)}\right]}_{5\times 6}\left(t=1,2,3\right)$ with linguistic scales (See Table 2, Table 3, Table 4).

**Table 2:** Linguistic scales from $UD_{1}$.

	UG_1_	UG_2_	UG_3_	UG_4_	UG_5_	UG_6_
UA_1_	UW	UVW	UM	UM	UT	UVW
UA_2_	UT	UVT	UM	UVT	UM	UVW
UA_3_	UVW	UM	UVT	UT	UW	UM
UA_4_	UM	UVT	UVW	UVW	UW	UVT
UA_5_	UVW	UVW	UVT	UM	UW	UVT

**Table 3:** Linguistic scale from $UD_{2}$.

	UG_1_	UG_2_	UG_3_	UG_4_	UG_5_	UG_6_
UA_1_	UM	UT	UVW	UW	UM	UW
UA_2_	UW	UVW	UVT	UT	UVW	UT
UA_3_	UM	UW	UT	UM	UVT	UT
UA_4_	UW	UVW	UT	UM	UT	UM
UA_5_	UVT	UVT	UM	UW	UVT	UM

**Table 4:** Linguistic scale from $UD_{3}$.

	UG_1_	UG_2_	UG_3_	UG_4_	UG_5_	UG_6_
UA_1_	UW	UM	UVT	UVW	UVW	UT
UA_2_	UM	UT	UVW	UW	UM	UVW
UA_3_	UW	UVW	UVT	UM	UM	UT
UA_4_	UVT	UVW	UM	UVT	UVT	UM
UA_5_	UVW	UVT	UM	UT	UVW	UW

Then according to q-ROFWA technique, the $UR={\left[UR_{ij}\right]}_{5\times 6}$ is managed (See Table 5).

Step 3 Conduct the weight numbers (Table 6):

**Table 6:** Attribute weight.

	UG_1_	UG_2_	UG_3_	UG_4_	UG_5_	UG_6_
uw	0.1952	0.2571	0.1939	0.0716	0.1307	0.1515

Step 4 Conduct the $UR^{+}=\left(UR_{j}^{+}\right)$ (Table 7):

**Table 7:** The ideal alternative $UR^{+}=\left(UR_{j}^{+}\right)\left(j=1,2,\cdots ,6\right)$.

	UG_1_	UG_2_	UG_3_
$UR^{+}$	(0.8214,0.3904)	(0.5219,0.5089)	(0.4603,0.5546)

	UG_4_	UG_5_	UG_6_
$UR^{+}$	(0.8436,0.2547)	(0.7365,0.3413)	(0.7312,0.4547)

Step 5 Compute the $WPRJ\left(UR_{i},UR^{+}\right)$ and $WBPRJ\left(UR_{i},UR^{+}\right)$ between each computer network systems $UR_{i}$ and $UR^{+}$ according to Eqs. (13) and (19) (See Table 8).

**Table 8:** The $WPRJ\left(UR_{i},UR^{+}\right)$ and $WBPRJ\left(UR_{i},UR^{+}\right)$.

	$WPRJ\left(UR_{i},UR^{+}\right)$	$WBPRJ\left(UR_{i},UR^{+}\right)$
UA_1_	0.4994	0.5121
UA_2_	0.8622	0.9094
UA_3_	0.6676	0.7099
UA_4_	0.5518	0.5961
UA_5_	0.7826	0.8169

Step 6 The order is conducted in line with Table 8 (See Table 9).

**Table 9:** The order for power grid enterprises.

	$WPRJ\left(UR_{i},UR^{+}\right)$	$WBPRJ\left(UR_{i},UR^{+}\right)$
UA_1_	5	5
UA_2_	1	1
UA_3_	3	3
UA_4_	4	4
UA_5_	2	2

**Table 5:** The $UR={\left[UR_{ij}\right]}_{5\times 6}$.

	UG_1_	UG_2_	UG_3_
UA_1_	(0.5089,0.46013)	(0.4216,0.6093)	(0.3704,0.7208)
UA_2_	(0.6876,0.3703)	(0.2879,0.7326)	(0.4603,0.5546)
UA_3_	(0.3119,0.7603)	(0.4702,0.7205)	(0.3879,0.6436)
UA_4_	(0.8214,0.3904)	(0.4883,0.5127)	(0.4121,0.6436)
UA_5_	(0.5218,0.4547)	(0.5219,0.5089)	(0.3326,0.7089)

	UG_4_	UG_5_	UG_6_
UA_1_	(0.5803,0.4213)	(0.5432,0.4925)	(0.5654,0.5809)
UA_2_	(0.7132,0.4436)	(0.6216,0.4554)	(0.7312,0.4547)
UA_3_	(0.3768,0.7214)	(0.5609,0.5123)	(0.4659,0.5214)
UA_4_	(0.8436,0.2547)	(0.7365,0.3413)	(0.6784,0.3325)
UA_5_	(0.5645,0.4436)	(0.3503,0.7223)	(0.4769,0.7108)

Finally, the order is obtained: $UA_{2}≻UA_{5}≻UA_{3}≻UA_{4}≻UA_{1}$, and the optimal power grid enterprise is $UA_{2}$.

### 5.2 Comparative analysis

**Fig. 1:**
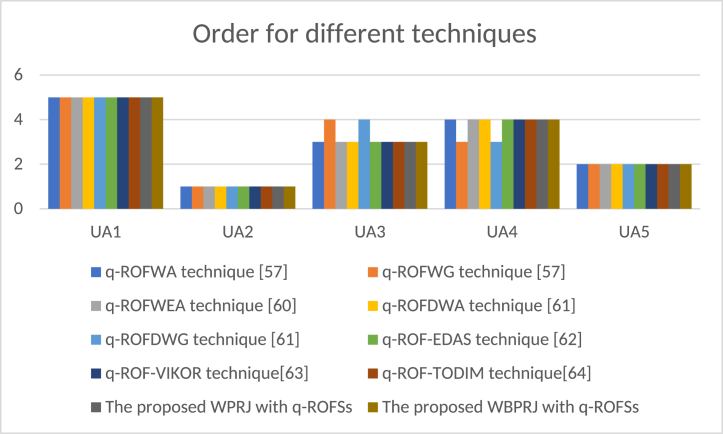
Order for different techniques.

Then, the proposed techniques is compared with q-ROFWA technique [58], q-ROFWG technique [58], q-ROFWEA technique [61], q-ROFDWA technique [62], q-ROFDWG technique [62], q-ROF-EDAS technique [63], q-ROF-VIKOR technique [64] and q-ROF-TODIM technique [65]. The comparative analysis is managed in Table 10 and Fig. 1.

**Table 10:** Order for different decision techniques.

Techniques	Order
q-ROFWA technique [58]	$UA_{2}≻UA_{5}≻UA_{3}≻UA_{4}≻UA_{1}$
q-ROFWG technique [58]	$UA_{2}≻UA_{5}≻UA_{4}≻UA_{3}≻UA_{1}$
q-ROFWEA technique [61]	$UA_{2}≻UA_{5}≻UA_{3}≻UA_{4}≻UA_{1}$
q-ROFDWA technique [62]	$UA_{2}≻UA_{5}≻UA_{3}≻UA_{4}≻UA_{1}$
q-ROFDWG technique [62]	$UA_{2}≻UA_{5}≻UA_{4}≻UA_{3}≻UA_{1}$
q-ROF-EDAS technique [63]	$UA_{2}≻UA_{5}≻UA_{3}≻UA_{4}≻UA_{1}$
q-ROF-VIKOR technique [64]	$UA_{2}≻UA_{5}≻UA_{3}≻UA_{4}≻UA_{1}$
q-ROF-TODIM technique [65]	$UA_{2}≻UA_{5}≻UA_{3}≻UA_{4}≻UA_{1}$
The proposed WPRJ with q-ROFSs	$UA_{2}≻UA_{5}≻UA_{3}≻UA_{4}≻UA_{1}$
The proposed WBPRJ with q-ROFSs	$UA_{2}≻UA_{5}≻UA_{3}≻UA_{4}≻UA_{1}$

Upon analyzing Tables 10, it can be deduced that the arrangement of these research techniques exhibits slight variations. Nevertheless, it is worth noting that these techniques yield identical optimal power grid enterprises and worst power grid enterprises. This compellingly demonstrates the efficacy of proposed techniques. Thus, the major advantages of the proposed techniques with q-ROFSs are outlined: (1) the proposed projection measure techniques with q-ROFSs not only grasps the uncertainty in MAGDM, but also grasps the psychological behavior during the risk assessment of “dual carbon” audit in power grid enterprises. (2) the proposed projection measure techniques with q-ROFSs analyze the behavior of the projection measure and q-ROFSs when they are combined. (3) human opinions with uncertainties are conducted strongly by employing q-ROFSs for challenging issues in MAGDM.

## 6 Conclusion

Achieving carbon peak and carbon neutrality holds immense importance in the overall and long-term strategies of economic and social development. To effectively implement the “dual carbon” policy, it is crucial for all industries within society to collaborate, adopt a comprehensive approach, and consistently promote its implementation. Furthermore, there is a need for extensive research aimed at enhancing the relevant regulations and systems pertaining to “dual carbon” auditing. This includes fostering the coordinated development of carbon and electricity markets and reinforcing the application of “dual carbon” audit evaluation results. One of the challenges in power grid enterprises is conducting risk assessments for “dual carbon” audits, which can be categorized as MAGDM problems. This study explores four types of projection measures using q-ROFNs and applies two weighted techniques to manage MAGDM. Additionally, a numerical example is presented to assess the risks associated with “dual carbon” audits in power grid enterprises, followed by a comparative analysis to validate the effectiveness of the developed techniques. The major contributions of this research are outlined: (1) Utilizing the entropy technique to determine weight values in accordance with SN and AN; (2)Implementing two weighted projection techniques to address MAGDM issues under q-ROFSs; (3) Demonstrating the application of developed weighted projection techniques under q-ROFSs through numerical example concerning risk assessment of “dual carbon” audits in power grid enterprises; (4) Conducting comprehensive comparative studies with existing techniques to showcase the efficiency of proposed approaches.

This study conducted in-depth research and introduced two weighted projection techniques for risk assessment of “dual carbon” audits in power grid enterprises under q-ROFSs. However, there are still several research limitations that need to be addressed in future research directions regarding the risk assessment of “dual carbon” audits in power grid enterprises. Firstly, the realization of carbon peak and carbon neutrality holds critical importance in the overall and long-term strategies of economic and social development. To enhance the implementation of the “dual carbon” policy, it is essential for all industries to collaborate, adopt a comprehensive approach, and continue promoting its adoption. Further research is required to improve the relevant regulations and systems of “dual carbon” auditing, facilitate the coordinated development of carbon and electricity markets, and strengthen the application of “dual carbon” audit evaluation results. Secondly, in our future research directions, it is recommended to integrate the weighted projection techniques with the risk assessment of “dual carbon” audits in power grid enterprises, considering frameworks such as the RANCOM model [66], CRITIC model [[67], [68], [69]], COMET model [[70], [71], [72]], MAIRCA model [[73], [74], [75]] and QUALIFLEX model [[76], [77], [78]]. This integration would contribute to a more comprehensive and robust evaluation of the risk associated with “dual carbon” audits in power grid enterprises.

## Ethics declaration statement

The authors state that this is their original work, and it is neither submitted nor under consideration in any other journal simultaneously.

## Data availability

The data used to support the findings of this study are included within the article.

## Funding information

The work was supported by the humanities and social science key project of the Anhui Department of Education. The research on the development trajectory of “carbon audit” in the context of ecological civilization, under Item number 2022AH052942 and the humanities and social science key project of the Anhui Audit College. The research on the development trajectory of “carbon audit” in the context of ecological civilization, under Item number 2022sjkyxm004.

## CRediT authorship contribution statement

**Shengnan Pan:** Writing – original draft, Methodology.

## Declaration of competing interest

The authors declare that they have no known competing financial interests or personal relationships that could have appeared to influence the work reported in this paper.
